# Relationship between weather conditions and admissions for ischemic stroke and subarachnoid hemorrhage

**DOI:** 10.3325/cmj.2017.58.56

**Published:** 2017-02

**Authors:** Adam D. Tarnoki, Acar Türker, David L. Tarnoki, Mehmet S İyisoy, Blanka K. Szilagyi, Hoang Duong, Laszlo Miskolczi

**Affiliations:** 1 Department of Radiology and Oncotherapy, Semmelweis University, Budapest, Hungary; 2 Department of Radiology, Abant Izzet Baysal University, Training and Research Hospital, Bolu, Turkey; 3 Department of Medical Education and Informatics, Necmettin Erbakan University School of Medicine, Konya, Turkey; 4 Division of Neurosurgery, Memorial Regional Hospital, Hollywood, Florida, USA; 5 Department of Interventional Neuroradiology, Holy Cross Hospital, Fort Lauderdale, Florida, USA

## Abstract

**Aim:**

To assess impacts of different weather conditions on hospitalizations of patients with ischemic strokes and subarachnoid hemorrhages (SAH) in South Florida.

**Methods:**

Diagnostic data of patients with spontaneous SAH and strokes were recorded between June 2010 and July 2013. Daily synchronous forecast charts were collected from the National Weather Service and the whole data were matched prospectively. The incidence rate ratio (IRR) was calculated.

**Results:**

Increased incidence rate of ischemic stroke was consistent with the daily lowest and highest air pressure (IRR 1.03, *P* = 0.128 and IRR 0.98, *P* = 0.380, respectively), highest air temperature (IRR 0.99, *P* = 0.375), and presence of hurricanes or storms (IRR 0.65, *P* = 0.054). Increased incidence of SAH cases was consistent with daily lowest and highest air pressure (IRR 0.87, *P* < 0.001 and IRR 1.08, *P* = 0.019, respectively) and highest air temperature (IRR 0.98, *P* < 0.001). Presence of hurricanes and/or tropical storms did not influence the frequency of SAH. We found no relationship between the presence of fronts and the admissions for ischemic stroke or SAH.

**Conclusion:**

Higher number of ischemic stroke and SAH cases can be expected with the daily lowest and highest air pressure, highest air temperature. Presence of hurricanes or tropical storms increased the risk of ischemic stroke but not the SAH. These findings can help to develop preventive health plans for cerebrovascular diseases.

Occurrence of stroke has been related to various factors. The ten risk factors are associated with 90% of the risk of stroke are history of hypertension, current smoking, waist-to-hip ratio, diet risk score, regular physical activity, diabetes mellitus, alcohol intake, psychosocial stress and depression, cardiac causes and ratio of apolipoproteins B to A1 (1). Moreover, circadian variation has been also shown to have an important effect (2). Recently, more attention has been oriented toward the effect of weather conditions on stroke admissions (3-5). However, evidence of the impact of air temperature and pressure and the extreme weather conditions (tropical storms, hurricanes) on cerebrovascular morbidity is still quite limited and controversial. Therefore, the objective of this study was to assess impacts of air pressure, air temperature, presence of weather fronts (warm, cold, mixed), hurricanes/storms on hospitalizations with strokes in South Florida, where these extreme weather conditions are quite frequently present.

## METHODS

### Study design

We searched for relationship between occurrence of stroke and weather conditions. Hospital admissions due to spontaneous non-traumatic subarachnoidal hemorrhages (SAH) and ischemic strokes were collected between June 2010 and October 2011 from Memorial Regional Hospital, Hollywood, FL, USA and between November 2011 and July 2013 from the Holy Cross Hospital, Fort Lauderdale, Florida, USA, based on data of patients who underwent angiography and brain computed tomography (CT) studies. All subarachnoid hemorrhage cases undergo a cerebral angiogram in the hospital which is standard of care. We collected SAH data by going through the Interventional Neuroradiology Angiogram Case Log which was very similar in both hospitals to select those patients who had CT images to confirm the presence of ischemic stroke or SAH. CT has a “stroke alert log”. Since all stroke cases must undergo CT of the brain, stroke cases were collected by getting the “stroke alert log” from CT. Accordingly, SAH and ischemic stroke data were collected prospectively from the beginning of the study to complete the SAH and stroke case logs. Then, retrospectively the data were matched day by day. The selection of the two hospitals was based on their Comprehensive Stroke Center status and the workplace of the last author (LM). Holy Cross Hospital and Memorial Regional Hospital accounted for the largest number of ischemic stroke and SAH interventions between 2010 and 2013 in Broward County, respectively. These two hospitals are close to each other (15 miles) and therefore the two are affected by the same meteorological events.

Daily forecast charts were downloaded each day prospectively from the website of the Hydrometeorological Prediction Center – NOAA, National Weather Service (http://www.wpc.ncep.noaa.gov/archives/web_pages/sfc/sfc_archive.php and http://w2.weather.gov/climate/index.php?wfo=mfl) in Florida area (Fort Lauderdale, Florida, 33308 and in Hollywood, Florida, 33021, retrospectively). The following data were collected from the downloaded images and websites on a daily basis from Florida area: daily lowest (APlow), highest (APhigh), and mean (APmean) air pressure, presence of high (Phigh), low (Plow), or no (Pneither) atmospheric pressure areas, the daily lowest (Templow), highest (Temphigh), and mean air temperatures, presence of no, cold, warm or mixed fronts, presence of hurricanes and tropical storms. All these conditions were labeled on the weather map by the weather service. Hurricane was defined by a violent, tropical, cyclonic storm of the western North Atlantic, having wind speeds of or in excess of 74 miles per hour (6). Tropical storm was referred to a condition with strong winds of over 39 miles per hour which is less than hurricane intensity (6). Weather front was defined if the front end or advancing edge of an air mass replaces the air mass that is over a specific region (6). For example, a cold weather front was referred as the changeover region where a cold air mass is replacing a warmer air mass. A high-pressure area was defined as a region where the atmospheric pressure at the surface of the planet is greater than its surrounding environment. Since a total of 63 days meteorological data were unavailable due to technical reasons, they were excluded from the final statistical analysis.

Each admission of ischemic stroke and/or SAH cases was expressed as occurrence number per day. In case of days in which no cerebrovascular events were reported, the incidence of ischemic stroke and/or SAH was recorded as zero.

IRB approval was not required due to the retrospective nature of this study. No patient information was collected, only the number of cases occurring on a certain day.

### Statistical analysis

Front and pressure variables were regarded as categorical variables with neither being the reference category. Because of over dispersion in data and many zeroes in the dependent variables, first zero-inflated poisson regression models and latterly negative binomial regression models were used for statistical evaluation. Zero-inflated models fit slightly better than negative binomial models while negative binomial models were more parsimonious. Therefore, we preferred negative binomial models consequently. Incidence rate ratio (IRR) values indicate that a one unit increase in the corresponding independent variable increases/decreases frequency of SAH or ischemic stroke. Statistical analysis was performed with Stata version 13 (StataCorp, College Station, Texas, USA) and R 3.1.2 (R Development Core Team 2015) with package pscl. *P* < 0.05 was considered statistically significant.

## RESULTS

### Descriptive analysis

**Figure 1:**
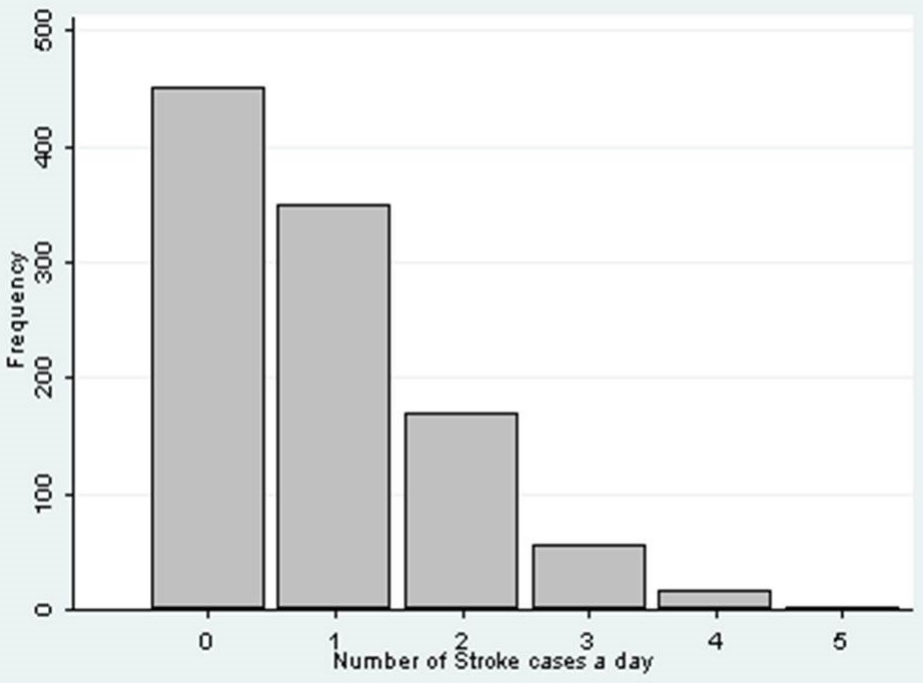
Frequency of ischemic stroke cases a day.

**Figure 2:**
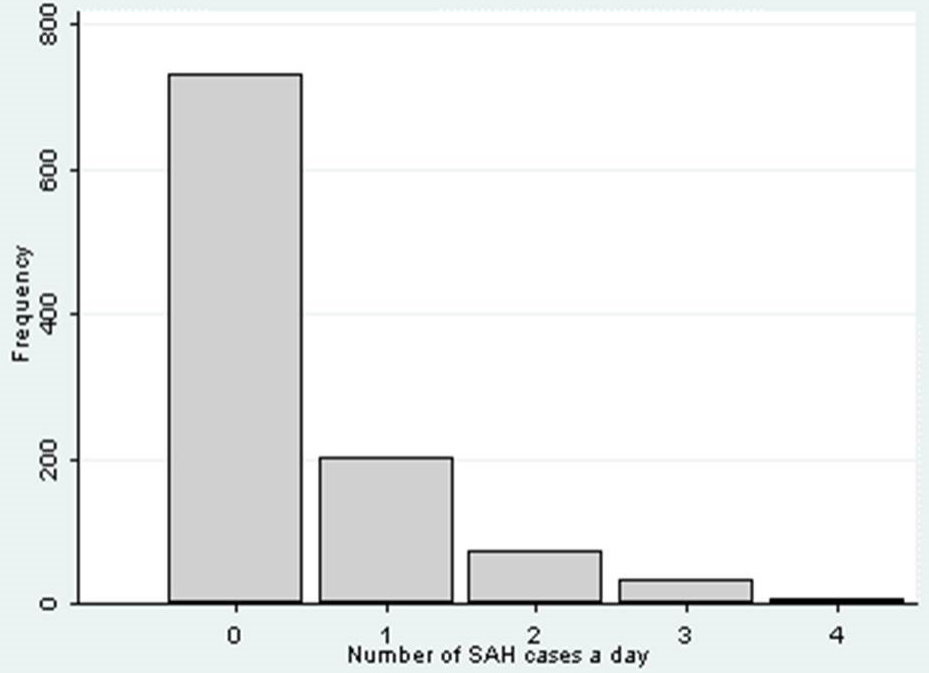
Frequency of for subarachnoid hemorrhage (SAH) cases a day.

Front and pressure variables were recorded and analyzed (Table 1 and Table 2). In the study period of 1045 days, 937 ischemic stroke cases happened in 594 days (Figure 1) and 473 SAHs happened in 314 days (Figure 2). Six days with a mixed air pressure (high and low pressure areas in the same day) were excluded from the analysis.

**Table 1:** Descriptive analysis of the weather sample in the study period

Variable* (unit)	Mean (standard deviation)	Min-max range
APlow (mb)	1015.52 (4.29)	995 – 1028
APhigh (mb)	1018.94 (4.33)	1004 – 1032
APmean (mb)	1017.24 (4.22)	1000 – 1030
Templow (F)	17.00 (14.07)	0 – 82.5
Temphigh (F)	68.82 (12.98)	29 – 88

*APlow, daily lowest air pressure, APhigh, daily highest air pressure, APmean, daily mean air pressure, Templow, daily lowest air temperature, Temphigh, daily highest air temperature.

**Table 2:** Weather characteristics in the study period

Weather variable	No. (%) of days
Air pressure	
normal	620 (63.01)
lowest	97 (9.86)
highest	267 (27.13)
Weather front	
no front	571 (58.15)
warm front	12 (1.22)
cold front	182 (18.53)
mixed front	217 (22.10)
Hurricane days*	
no	974 (98.38)
yes	16 (1.62)
Tropical storm days^†^	
no	935 (94.44)
yes	55 (5.56)

*Hurricane day was defined the day of the investigation when a violent, tropical, cyclonic storm of the western North Atlantic, having wind speeds of or in excess of 72 miles per hour (32 m/s) effected the Florida state, including the Broward county where the sampling hospitals are located. Hurricanes Karl (2010 September), Irene (2011 August), Cina (2011 October), Isaac (2012 August), Sandy (2012 October), Barbara (2013 May) and Andrea (2013 June) affected the region in the observation period.

†Tropical storm day was defined the day of the investigation when a weather condition with strong winds of over 39 miles (63 km) per hour which is less than hurricane intensity effected the Florida state, including the Broward county where the sampling hospitals are located.

### Individual associations with ischemic stroke

We searched for individual associations with number of ischemic stroke cases. Negative binominal regression was used instead of zero inflated poisson regression since this model is parsimonious, while zero inflated model slightly fits better. In these models, APlow (*P* = 0.025), presence of hurricanes or storms (*P* = 0.011), Plow (*P* = 0.056) and Phigh (*P* = 0.003) in reference to Pneither, APmean (*P* = 0.037) were found significant predictors individually. APhigh (*P* = 0.054), Templow (*P* = 0.100), Temphigh (*P* = 0.065), presence of tropical storms (*P* = 0.075), hurricanes (*P* = 0.065), warm fronts (*P* = 0.430), cold fronts (*P* = 0.309), mixed fronts (*P* = 0.661) in reference to neither were not significant.

### Negative binominal regression models for ischemic stroke cases

**Figure 3:**
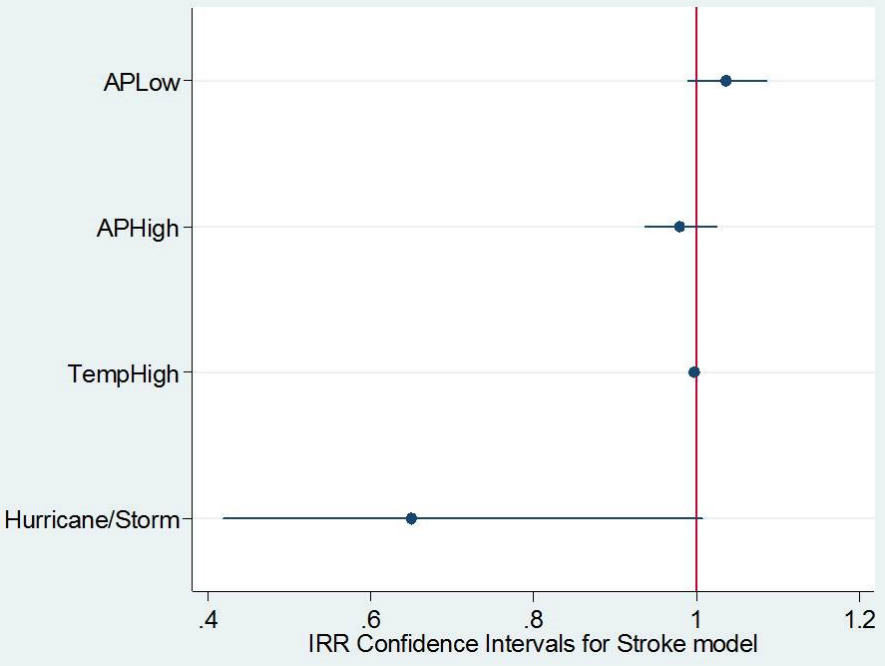
Incidence rate ratio (IRR) values with confidence intervals for ischemic stroke model indicating that ischemic stroke cases were predicted by daily lowest air pressure (APlow), daily highest air pressure (APhigh), daily highest air temperature (Temphigh), and presence of hurricanes/storms.

Ischemic stroke cases were predicted by APlow, APhigh, Temphigh, and presence of hurricanes or storms (Figure 3). The IRR values for APlow, APhigh, Temphigh, and presence of hurricanes or storms were 1.03 (*P* = 0.128), 0.98 (*P* = 0.380), 0.99 (*P* = 0.375), and 0.65 (*P* = 0.054), respectively.

### Individual associations with SAH cases

Individual negative binominal regression models with SAH being dependent variable yielded the following significant associations for the other variables in concern: Temphigh (*P* = 0.005), APhigh (*P* = 0.005), APmean (*P* = 0.041), these were found significant predictors. Templow (*P* = 0.743), APlow (*P* = 0.221), warm front (*P* = 0.913), cold front (*P* = 0.978), mixed front (*P* = 0.937) (front neither being reference category), Phigh (*P* = 0.661), Plow (*P* = 0.900) (P neither being reference category), presence of hurricanes (*P* = 0.312), tropical storms (*P* = 0.611) or either of them (*P* = 0.941) were not found significant.

### Negative binominal regression models for SAH cases

**Figure 4:**
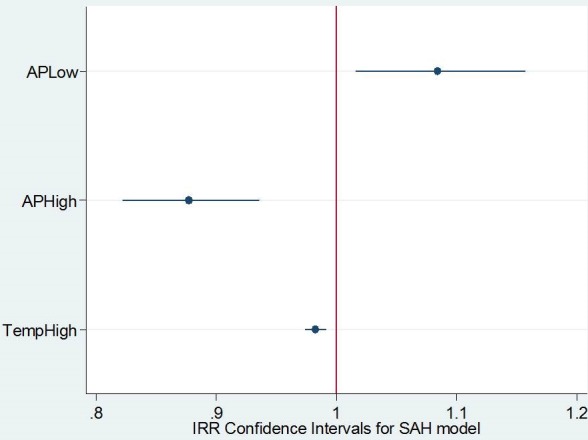
Incidence rate ratio (IRR) values with confidence intervals for subarachnoid hemorrhage (SAH) model indicating that SAH cases were predicted by daily lowest air pressure (APlow), daily highest air pressure (APhigh), and daily highest air temperature (Temphigh).

Based on above findings two final models were formed. Plain negative binominal regression models were applied because there was not much statistically difference between zero inflated negative binominal regression model and plain negative binominal regression model. In the first model, SAH cases were predicted by APlow, APhigh and Temphigh (Figure 4). The following IRR values were significant: APhigh IRR = 0.87 (*P* < 0.001), APlow IRR = 1.08 (*P* = 0.019) and Temphigh IRR = 0.98 (*P* < 0.001).

## DISCUSSION

To our knowledge, this is the first study which has demonstrated that ischemic stroke cases were associated with low and high air pressure, high air temperature and interestingly, presence of hurricanes or tropical storms. In addition, subarachnoid hemorrhages were associated with daily lowest and highest air pressure, daily highest air temperature, but the presence of hurricanes or tropical storms did not influence their frequency. We found no relationship between the presence of fronts and the admissions for ischemic stroke or SAH.

In previous works several meteorological variables, such as variations in temperature, atmospheric pressure, humidity, and the lunar cycle have been linked to an altered incidence of cerebrovascular disease, however, the findings have been inconsistent (7-10). A large nationwide study demonstrated that decreased sunlight and lower relative humidity are also related to admission for SAH from ruptured cerebral aneurysms (10). A South African two-year long retrospective analysis found a relationship between aneurysm rupture and a change in barometric mean pressure >10 hectopascals from the previous day, which is in line with our findings (7). Another study confirmed the seasonal fluctuation between seasonal and climatic conditions and SAH rupture in Connecticut State for the fiscal years 1981, 1983, 1985, 1987, 1988, and 1989 (11). A link between air temperature at onset of SAH has not been proven in a previous study (12). In our study, we investigated not only the mean air temperature but also the highest daily air temperature which could serve as a possible explanation why we found a relationship. Favoring our results in case of SAH, a recent Korean study suggested that the daily temperature swings may influence the risk of spontaneous intracerebral hemorrhage (13). Studies performed in Siberia and in France also failed to report an association between any one of the weather parameters studied and the occurrence of SAH (14,15). We suspect that the lack of warmer weather conditions in these regions compared to Florida limited those studies to achieve a link between air temperature and the occurrence of SAH in contrast to our findings. It has been postulated that these external atmospheric factors may cause hormonal and homeostatic changes that impact the risk of rupture of cerebral aneurysms. For instance, these factors are most likely blood pressure related in SAH cases and probably stress related in ischemic stroke cases. Stress induces extra arrhythmia or other rhythm changes or variations, resulting in emboli of cardiac origin. Additional research is needed to confirm and further understand these relationships (10).

Our work also focused on the effects of weather conditions on ischemic strokes as well, not only subarachnoid hemorrhages. It is well known that hypertension, smoking, diabetes, blood pressure, obesity, hypercholesterolemia, physical inactivity, dietary factors, atrial fibrillation or other arrhythmias are well known risk factors for stroke. There are few studies which revealed the relationship between meteorological variables and stroke. For example, a previous paper assessed the impacts of air temperature, barometric pressure and geomagnetic activity on hospitalizations with myocardial infarctions and brain strokes between 1992 and 2005 (5). The number of strokes increased with temperature and daily temperature range, associations with low pressure and falling pressure were observed which is in line with our observations since we also reported that stroke cases are predicted by daily lowest - and also highest - air pressure and highest air temperature. Han et al. recently demonstrated distinct patterns of seasonal and monthly variation in the stroke incidence and its subtypes through consideration of the meteorological and air pollution parameters in nine year term in Seoul, South Korea (3). They found that the mean temperature was positively correlated with stroke which is in line with our findings (3). On contrary, a recent Turkish retrospective study investigated the association between certain weather patterns (daily temperature, humidity, wind speed, air pressure) and year long stroke admissions (4) and did not find any association between overall admissions due to stroke and meteorological parameters (4). Interestingly, our study reported that the weather fronts had effect neither on SAH nor on ischemic stroke admissions which phenomenon has never been published earlier to our knowledge.

The main novelty of our study is the investigation of hurricanes and tropical storms on ischemic stroke and SAH admissions. We demonstrated a relationship between ischemic stroke and the presence of hurricanes or tropical storms but not with SAH. Only one study has assessed this phenomenon in the USA, when Hurricane Sandy made huge destruction in New Jersey on October 29, 2012. This American study aimed to impact the incidence of cardiovascular events during this extreme weather change in New Jersey in the following two weeks (16). The results showed that stroke incidence increased by 7% and the incidence of stroke and 30-day mortality increased as well (16). In the current study, we confirmed that this is not a unique case but that the increased ischemic stroke incidence relates to hurricanes and tropical storms in general. The mechanism suggested by other studies included arrhythmias and increased stress, linked to platelet activation, increased physical activity, altered blood rheology during natural disasters or extreme weather events (16,17). It is likely that an interaction between these factors played a major role in morbidity following these events (16). In addition, the final outcome could be also impacted by the condition whether those patients had the same level of care or decreased level of hospital care.

These findings could stimulate further studies to investigate whether patients could benefit from the increased surveillance in radiological units (CT, intervention for transcatheter intraarterial thrombolysis) in the period of extreme weather conditions in order to decrease mortality risk and delay in elective procedures. For example, storm preparations could include steps like stocking up extra thrombolytic drugs (eg, tPA), thrombectomy catheters, increase the number of specialists (neurosurgeons, anesthesiologists, radiologists etc.) in order to be able to cope the potentially increased stroke volumes. Given that medical facilities develop emergency preparedness plans for future events, these preparations should be facilitated in advance and strategies to repurpose underutilized resources from a reduction in elective procedures should be taken into consideration (16).

Several limitations are inherent to this study. First, some clinical variables and characteristics such as risk factors for ischemic stroke and SAH, including previous/present medication and/or comorbidities (such as diabetes mellitus, hypertension, amyloid angiopathy, prior SAH and/or ischemic stroke history) that might directly affect the occurrence and precise onset time of the infarcts. In addition, the age, gender was also not recorded which limited us to investigate their effects. Of note, south Florida is well equipped with comprehensive stroke centers, and patients seldom need to travel farther than 30 minutes in order to get to an appropriate hospital, indicating a 30 minutes ambulance time which translates to a 30 mile radius. Essentially, the size of the study population area is not larger than Broward county itself. To this end, the major strength of our study is the location of Florida, which allowed the assessment of extreme weather conditions and higher overall temperatures which has been lacking in the literature so far. Further studies should investigate whether the stroke is more frequent in regions with cold weather.

In conclusion, ischemic strokes and SAHs were predicted by low and high air pressure and high air temperature in South Florida. In addition, presence of hurricanes or tropical storms increased the risk of ischemic stroke but not the SAH. No relationship between the presence of fronts and the admissions for ischemic stroke or SAH was detected. These findings can help to develop preventive health plans for cerebrovascular diseases.
